# Validation and refinement of cropland map in southwestern China by harnessing ten contemporary datasets

**DOI:** 10.1038/s41597-024-03508-5

**Published:** 2024-06-22

**Authors:** Yifeng Cui, Jinwei Dong, Chao Zhang, Jilin Yang, Na Chen, Peng Guo, Yuanyuan Di, Mengxi Chen, Aiwen Li, Ronggao Liu

**Affiliations:** 1 Institute of Geographic Sciences and Natural Resources Research, Chinese Academy of Sciences, Beijing, 100101 China; 2 University of Chinese Academy of Sciences, Beijing, 100049 China; 3 Department of Civil and Environmental Engineering, Massachusetts Institute of Technology, Cambridge, MA 02139 USA; 4 Department of Civil and Environmental Engineering, National University of Singapore, Singapore, 117576 Singapore; 5 College of Grassland Science and Technology, China Agricultural University, Beijing, 100193 China; 6 Institute of Remote Sensing and Geographic Information System, School of Earth and Space Sciences, Peking University, Beijing, 100871 China; 7 Faculty of Science and Engineering, University of Nottingham Ningbo China, Ningbo, 315100 China; 8 College of Resources, Sichuan Agricultural University, Chengdu, 611130 China

**Keywords:** Agroecology, Sustainability

## Abstract

Accurate cropland map serves as the cornerstone of effective agricultural monitoring. Despite the continuous enrichment of remotely sensed cropland maps, pervasive inconsistencies have impeded their further application. This issue is particularly evident in areas with limited valid observations, such as southwestern China, which is characterized by its complex topography and fragmented parcels. In this study, we constructed multi-sourced samples independent of the data producers, taking advantage of open-source validation datasets and sampling to rectify the accuracy of ten contemporary cropland maps in southwestern China, decoded their inconsistencies, and generated a refined cropland map (Cropland_Syn_) by leveraging ten state-of-the-art remotely sensed cropland maps released from 2021 onwards using the self-adaptive threshold method. Validations, conducted at both prefecture and county scales, underscored the superiority of the refined cropland map, aligning more closely with national land survey data. The refined cropland map and samples are publicly available to users. Our study offers valuable insights for improving agricultural practices and land management in under-monitored areas by providing high-quality cropland maps and validation datasets.

## Background & Summary

Croplands feed human beings and sustain life on Earth. Since a billion people are still facing hunger, cropland plays an irreplaceable role in meeting the world’s increasing future food security and sustainability needs^1^. In addition, cropland may have significant impacts on ecosystems^2^. For example, the process of agricultural intensification and expansion may encroach on protected areas or forests, leading to the destruction of species’ habitats and extinction^3,4^ or affects the process of terrestrial carbon cycle^5,6^. Meanwhile, post-agricultural landscapes, represented by cropland abandonment, continuously impact soil organic carbon sequestration in the context of climate change^7^. The United Nations’ 2030 Sustainable Development Goals (SDGs) targets therefore call for national cooperation and policies to improve food security (SDG 2), protect ecosystems (SDG 15), and combat climate change (SDG 13)^8^. In this regard, timely, accurate, and affordable spatiotemporal cropland datasets are the basis for achieving these goals^9,10^.

Satellite data with spatiotemporally consistent earth observation (EO) have enabled agricultural monitoring from regional to global scales^11,12^. In the past four decades, multiple research teams have delivered hundreds of approaches to produce land cover datasets worldwide, most of which include the category of cropland^13–15^. The most remarkable advance over time has been the improvement in spatial resolution^16^. In the 2010s, the spatial resolution of EO-based land use products experienced a great shift from coarse to medium level, reaching 30 m with the freely accessible Landsat archives from the United States Geological Survey (USGS)^13,17^. Subsequently, with the successive launches of higher spatial resolution satellite sensors (i.e., GaoFen^18^, Sentinel^19^ PlanetScope^20^), as well as the advances in cloud computing data processing techniques such as Google Earth Engine (GEE)^21^ and the iteration of machine learning algorithms^22^, it has become possible to conduct more detailed landscape-scale cropland mapping at 10-m or higher spatial resolution levels. Against this background, several research teams have popped up cropland-specific datasets and all-type land use and land cover (LULC) products with 30-m or higher spatial resolution since 2021^23,24^. Nonetheless, there is considerable variation in the portrayal of the amount and spatial extent of cropland among the different datasets, with the spatial resolution sharpening from hundreds of meters to mere tens of meters, as some factors that can be ignored at low resolutions would become the main signals^25^. Let alone the widely existing inconsistency of criteria, source of data, and classification methods, as well as the lack of independent quantitative assessment of these maps, poses challenges. This is especially true in the context of agricultural monitoring in areas with limited good observations^26^, high topographic relief, fragmented parcels, and fragile ecological environments like southwestern China, which constrains the in-depth applications of existing datasets^27–29^.

Previous studies have shown that the worldwide substantial inconsistencies lie in three newly published LULC datasets for 2020, including ESA WorldCover, ESRI’s Land Cover, and Google Dynamic World^30^. In the sub-globe scale, there are also reported cases of consistency assessment of multi-class land cover products^31–33^ or thematic maps^34,35^ that point to the aforementioned issues. For example, it was found that the percentage of inconsistency between five widely used cropland datasets for Africa is more than 1/3^36,37^. An evaluation from Gao, *et al*.^38^ in Europe demonstrated higher consistency and accuracy for cropland and forest categories in three 30-m LULC maps, but lower consistency in the mountainous areas. These studies collectively show that global precision does not necessarily indicate better demonstrate local performance at the regional level^39^. A similar situation was reported in the forest evaluation for seven global land cover datasets of 2010^34^ and six cropland maps of 2020^40^ in China, as well as the accuracy quantification of six 30-m cropland datasets in circa 2015^41^. These studies have carried out assessment work on administrative scales such as national, provincial, and other scales, based on datasets updated in 2020 or earlier with spatial resolutions equal to or l coarser than 30 m. Since 2020, more than ten newly released and continuously updated datasets with cropland categories provide unprecedented detail of EO at 10-m or higher spatial resolution^24,42^. However, the consistency and accuracy of these newly published datasets have not been independently assessed and compared, especially in complex terrain and fragmented parcels where cropland mapping has historically been more challenging.

Here, we first constructed a validation dataset by integrating validation samples from publicly available datasets as well as stratified random sampling and generated a consistency map to quantify the spatial pattern of ten existing cropland datasets in southwestern China and then presented a refined dataset that provides an optimized distribution of cropland with a 30-m spatial resolution (Cropland_**Syn**_). This study details the production process of these datasets, including accuracy rectification of the existing cropland maps, decoding the inconsistencies, and generating the refined map. Our study provides a clear perspective for understanding the inconsistency of different cropland maps and generated a data-driven refined map to retrieve better the spatial extent and cropland area in southwestern China.

## Methods

### Study area

**Fig. 1:**
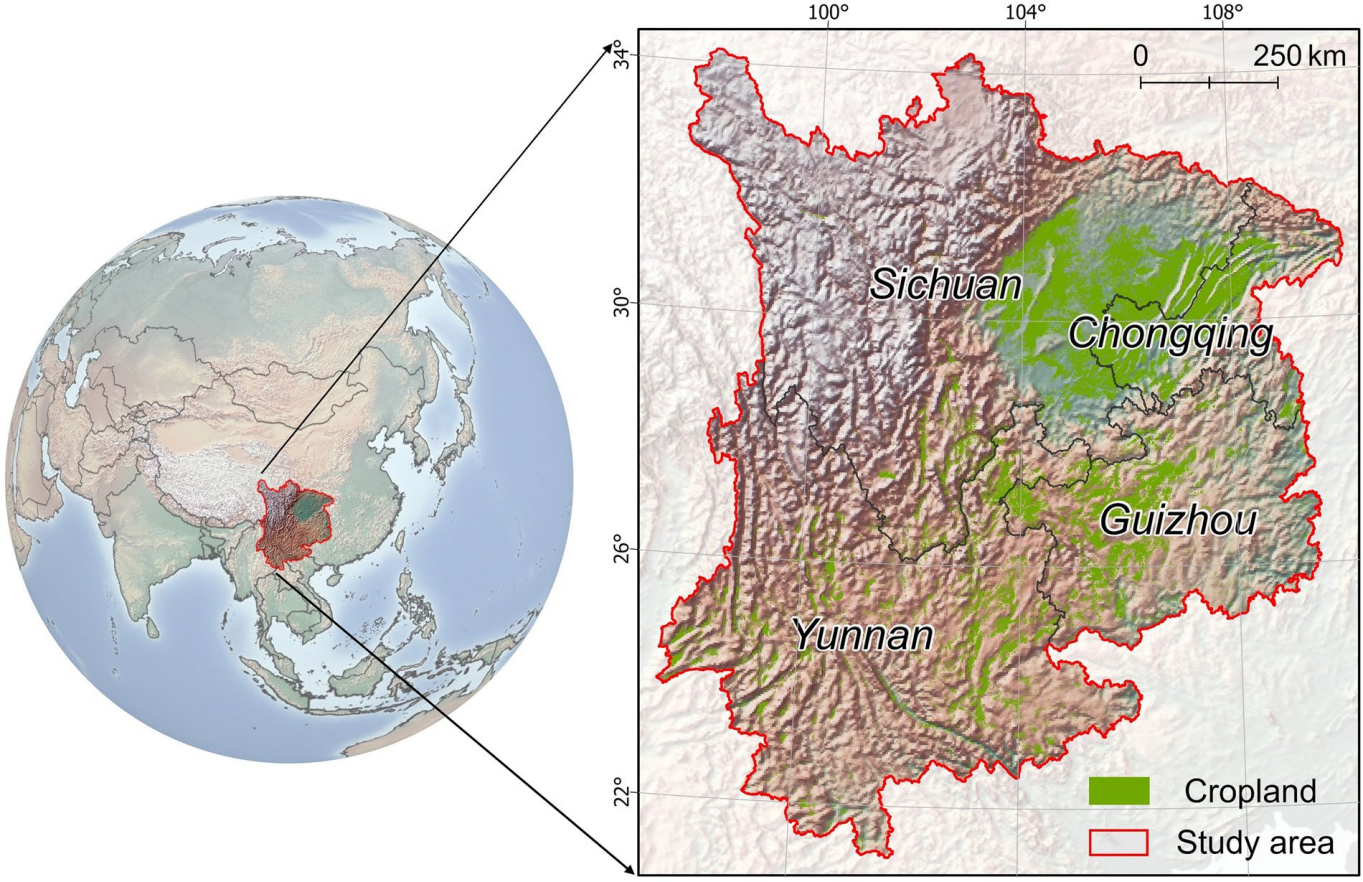
Location of southwestern China. The cropland layer overlaid in the right zoomed-in figure was derived from MCD12Q1 Version 06, available at https://lpdaac.usgs.gov/products/mcd12q1v006/.

The study area is located in southwestern China, with geographic coordinates ranging from 22°29′N to 34°21′N and 97°21′E to 111°47′E (Fig. 1). It encompasses four provincial administrative units: Chongqing, Sichuan, Guizhou, and Yunnan. This region is characterized by rich natural resources and diverse ecosystem types, featuring fragmented parcels and varied topography including plains, basins, hills, and mountains^43^. The study area is also home to approximately 200 million people and stands as one of China’s most important agricultural production areas. Cropland in this area is primarily located in basins and plains with rainy and cloudy climates, such as the Sichuan Basin and the Yunnan-Guizhou Plateau. Cropland is also cultivated in flat dams and river valleys, with the Sichuan Basin renowned as the “Breadbasket of Tianfu” (Fig. 1). Given the diversity of topography and the intensification of human-land conflicts, the accurate cropland map is of utmost significance in ensuring the sustainable management of cropland in southwestern China^44^.

### Cropland maps

#### Ten contemporary land cover/use maps for croplands

In this study, we analyzed ten of the latest published cropland maps after 2021^45–54^ to generate a refined cropland map. These maps are global or local-scale cropland thematic or LULC maps released in recent three years (2021–2023).Table S1 summarizes the general metadata of these maps, which span a range of geographic extent and various spatial resolutions. The accessibility of these cropland maps is demonstrated in Data Records.

##### Sino-LC1

The Sino-LC1 is China’s first national-scale land cover map with a spatial resolution of nearly 1 m^45^. It was established by using a low-cost deep learning-based framework and open-access data (including global land-cover (GLC) products, open street map (OSM), and Google Earth imagery). The dataset comprised 11 land cover types, of which cropland was labeled as 5. Due to the problem of updating and coverage of Google Earth’s data in China, the producers of Sino-LC1 used interpolation methods to fill gaps in data.

##### ESA WorldCover

The 10-m WorldCover product produced by the European Space Agency (ESA) provides free access to the 2020 global land cover map derived from Sentinel-1 and Sentinel-2 satellite data^46^. The WorldCover product comes with eleven land cover classes, aligned with UN-FAO’s Land Cover Classification System, and independently validated with a global overall accuracy of about 75%.

##### ESRI Land Cover

The ESRI Land Cover dataset is a global LULC map for 2020 and derived from ESA Sentinel-2 imagery at 10-m resolution^47^. It is a composite of LULC predictions for ten classes (where croplands are defined as human-planted/plotted cereals, grasses, and crops not at tree height; examples: corn, wheat, soy, fallow plots of structured land) of 2020. The ESRI Land Cover dataset was produced by a deep learning model (uses six bands of Sentinel-2 surface reflectance (SR) data: visible blue, green, red, near-infrared, and two shortwave infrared bands) and was trained using over 5 billion hand-labeled Sentinel-2 pixels, sampled from over 20,000 sites distributed across the world. The dataset achieves an overall accuracy of 86% for global validation.

##### Dynamic World

Dynamic World is a near real-time 10-m global LULC dataset, produced by deep learning on the Sentinel-2 Level1C remote sensing data from 2015 to the present, freely available through the Google Earth Engine and openly licensed^48^. It is the result of a partnership between Google and the World Resources Institute to produce a dynamic dataset of the physical material on the surface of the Earth. The dynamic world has three characteristics: near real-time, per-pixel probabilities across nine land cover classes and 10-m resolution. Dynamic World generates more than 5,000 images per day, and by utilizing a novel deep learning methodology based on Sentinel-2 top-of-atmosphere, thus can update global land cover data every 2–5 days (the specific revisit period depends on its position on earth). As the annual cropland map for 2020 was evaluated in this study, a composite method of majority in Earth Engine was performed for the Dynamic World dataset in the data pre-processing to generate the annual composite cropland map.

##### CRLC

The CRLC is the name of the framework cross-resolution national-scale land-cover^49^. This study used the CRLC to represent the 10-m resolution land-cover map. It was completed using the CRLC framework based on Sentinel-2 imagery and 30-m historical products (GlobeLand30-2010) and offers the possibility to update products quickly and efficiently globally. The dataset covers eight land-cover types, and the results show that the estimated user accuracy for cropland is 81.72% and the estimated producer accuracy is 81.64% with an estimated area of 1805.1 ± 56.6 10^3^ km2.

##### GlobeLand 30

GlobeLand 30 was developed by the National Geomatics Centre of China (NGCC) with 30-m spatial resolution, and it provides multi-temporal land cover images at 10-year intervals (2000/2010/2020)^50^. The data source comes from multi-spectral images, including Landsat TM and ETM+ multispectral images. Globeland 30 contains ten land cover types, and the overall accuracy of this data is 80.30%, with an overall accuracy of 82.39% within China. It was first released for open access and non-commercial utilization in 2014, and the version of 2020 was updated in 2021.

##### CLCD

The China land cover dataset (CLCD) is a Landsat-derived annual dataset processed on the Google Earth Engine platform. It contains annual land cover in China from 1990 to 2022 at 30-m spatial resolution^51^. For the processing, several temporal indicators were constructed using 335,709 Landsat images on Google Earth Engine and fed into a random forest classifier to obtain classification results. The overall accuracy of CLCD reached 79.31% based on 5,463 visually interpreted samples by the data producer.

##### GLC_FCS30

Global land-cover product with fine classification system (GLC_FCS30) version 2020 provides global fine-classified land cover products at 30-m spatial resolution using Landsat time-series imagery^52^. The GLC_FCS30 provided a time series dataset from 1985 to 2020 with a 5-year interval and utilized continuous Landsat imagery from the Google Earth Engine platform. In particular, GLC_FCS30 2020 is based on the 2015 version product, optimized by combining multi-source auxiliary datasets (e.g., the 2019-2020 Landsat SR data, Sentinel-1 SAR data, DEM terrain elevation data, global thematic auxiliary dataset) and a priori knowledge from experts (e.g., the new GLC_FCS30 2020 product has further improved the classification performance for cropland comparing with its predecessor GLC_FCS30 2015).

##### GLAD

The GLAD used in this study is the abbreviation for the Global cropland expansion in the 21^st^-century dataset produced by the Global Land Analysis & Discovery team, which represents a globally consistent cropland extent time-series at 30-m/pixel spatial resolution from 2000 to 2019^53^. Cropland mapping used the consistently processed Landsat satellite data with four-year intervals (2000–2003, 2004–2007, 2008–2011, 2012–2015, and 2016–2019)^55^. The cropland layer for each epoch is named after the last year of the product period (five maps in total, 2003, 2007, 2011, 2015, and 2019). Using a longer time interval of four years (rather than a single year) increases the available satellite imagery data within the time series. On the other hand, in this dataset, the fallow length is limited to four years for the cropland class (in each four-year interval, mapped an area as cropland if a growing crop was detected during any of these years), which can improve the representativeness of land surface phenology and the accuracy of cropland detection. Due to the lack of the latest version of 2019–2023, the 2019 version was used in the study.

##### CACD

China’s annual cropland dataset (CACD) is a 30-m annual cropland dataset of China from 1986 to 2021^54^. The dataset utilizes all available Landsat TM/ETM+/OLI Tier 1 SR imagery at a 30-m spatial resolution from 1986 to 2021. Annual cropland in this dataset is defined as a piece of land of 0.09 ha in minimum (minimum width of 30 m) that is sowed/planted and harvestable at least once within the 12 months after the sowing or planting date. The production of the dataset was applied using automated training sample generation, random forest supervised classification, and the LandTrendr temporal segmentation algorithm on the Google Earth Engine platform, enabling cost-effective monitoring of fine-resolution dynamics cropland identification.

#### Generation of binary cropland maps

**Fig. 2:**
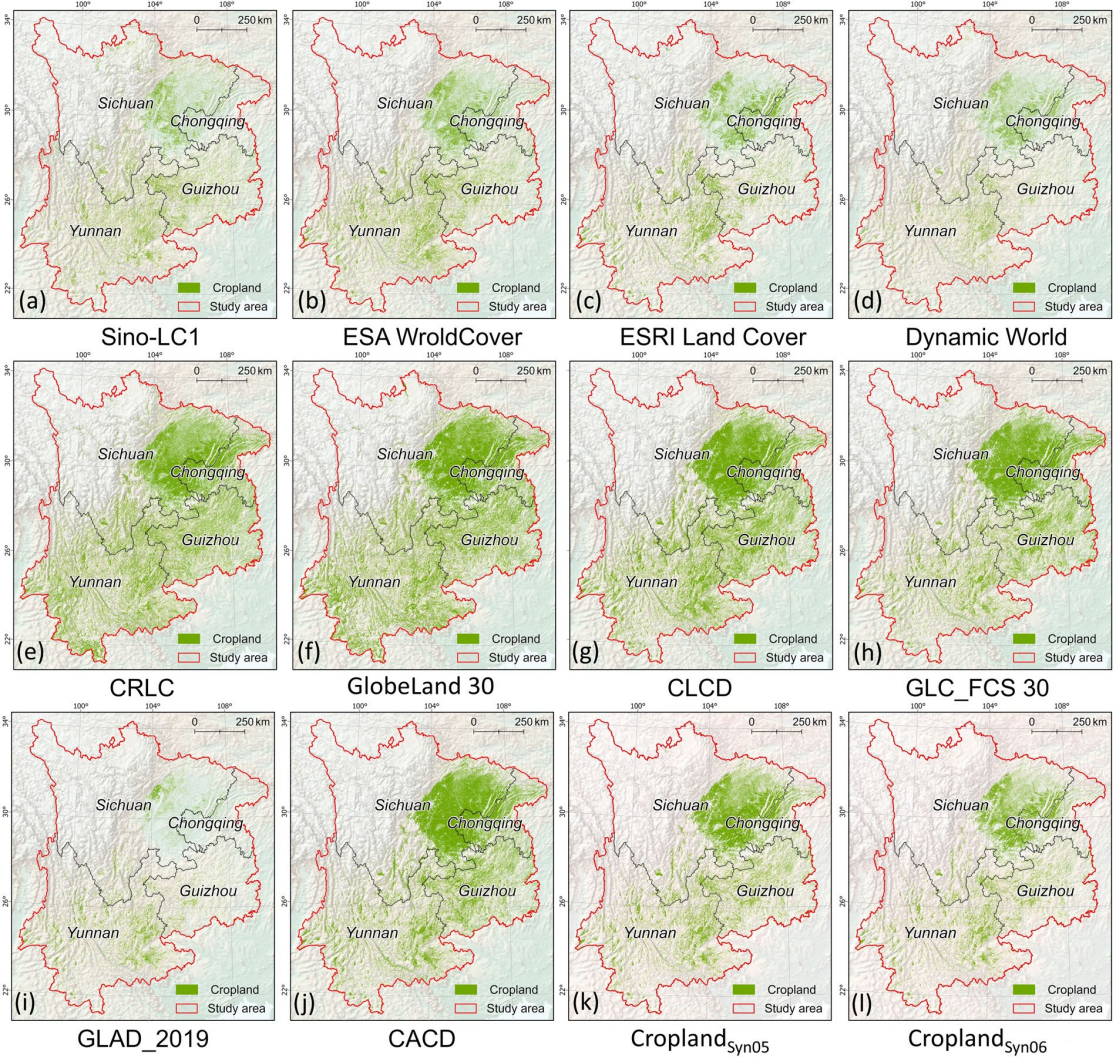
Illustration of cropland maps in this study. Ten existing cropland maps (**a**–**j**) and two refined maps with threshold 5 (**k**) and 6 (**l**), respectively. All the twelve maps are with 30-m spatial resolution.

According to the definition of cropland in each dataset (Table S1), the binary cropland maps were first extracted from the corresponding LULC maps in GEE and then clipped by the boundaries of southwestern China. All cropland datasets utilized in the study were converted to the Albers Equal Area Conic projected coordinate system (PCS) to facilitate area calculation and comparison. All maps were resampled to 30-m resolution using the nearest method and were batch-conducted using the ArcPy module in a Python environment. Following these pre-processing steps, the binary maps were generated with the pixel value of 1 for the cropland and 0 for the noncropland, respectively (Fig. 2).

### Accuracy assessments based on independent sample set

#### Generation of ground-truth sample independent from map producers

**Fig. 3:**
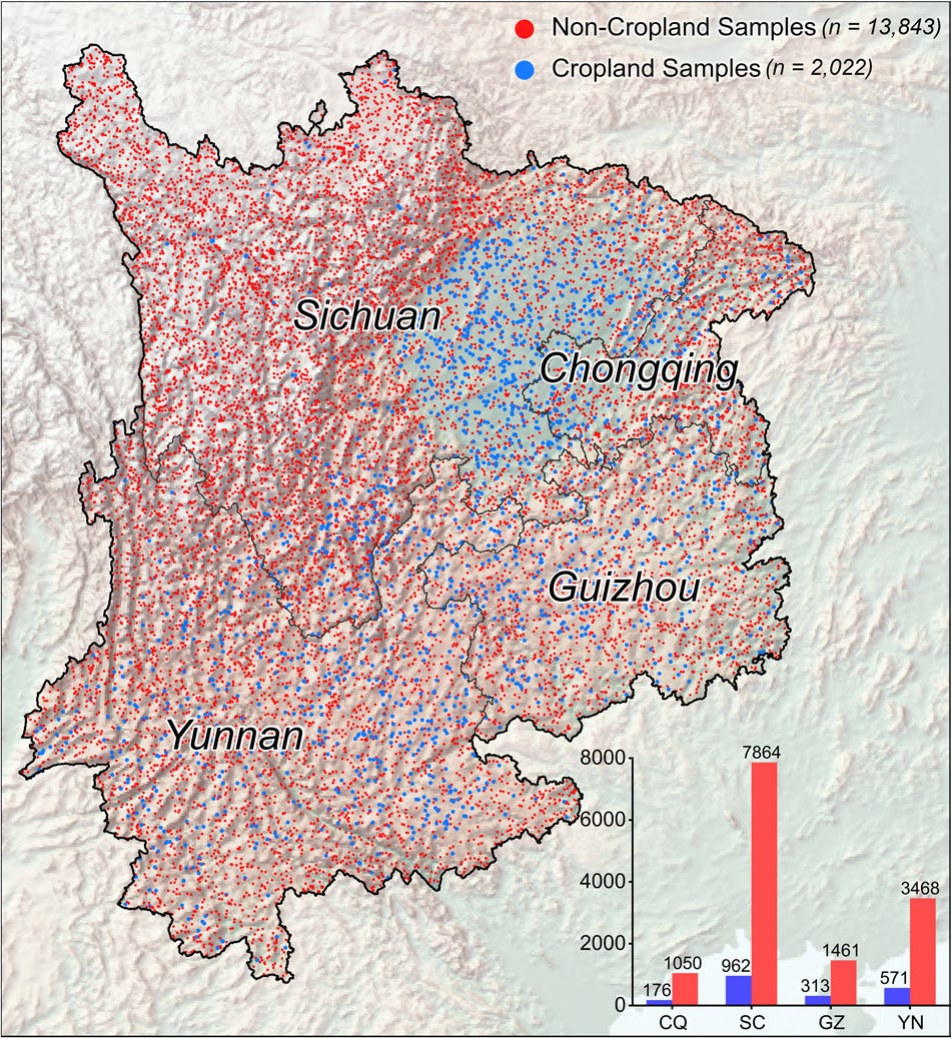
Spatial distribution of validation samples in southwestern China.

The quality of the reference sample is crucial for an accurate assessment, especially where global accuracy is known to be poorly characterized by local accuracy^56^. A ground-truth reference sample derived from a multi-sourced sample pool was constructed to rectify the regional accuracy of ten cropland maps in southwestern China. The sample set contains 15,865 ground-truth samples (2,022 for cropland and 13,843 for non-cropland category), with three properties: the latitude and longitude coordinates for geological position and the label of cropland (code: 1) and non-cropland (code: 0) attached to each item (Fig. 3).

Constructing a reference sample set integrates existing samples from public accessible libraries and the additional samples from stratified sampling-aided filed surveys^41^. The first part comes from the southwestern China subset of the sample pool in the Global Food Security-support Analysis Data (GFSAD)^57^, Annual Global Land Cover (AGLC)^58^ and Global Land Cover Estimation (GLanCE)^59^, with 1,691, 1,296, and 2,387 samples, respectively. These samples were randomly distributed across southwestern China. In the second part, we conducted a stratified random sampling with the strata defined by the proportion of the cropland consistency map. We generated a total of 10,491 sample points within the maximum cropland extent to increase the density of samples. Eventually, 15,865 ground-truth units were composited and distributed across southwestern China (within and outside the potential cropland extent) (Table 1). They then underwent cross-validation using Google Very High Resolution (VHR) images from around 2020, reviewed by two trained senior specialists individually, to ensure that the reference set is stable and representative.

**Table 1:** Composition of sample used for accuracy rectification in Southwestern China.

Sub-region	Admin Code	Source of sample				Summary		
		GFSAD	AGLC	BU Glance	Stratified random sampling	Cropland	Non-Cropland	Sum
Chongqing	500000	161	135	41	889	176	1050	1226
Sichuan	510000	735	517	2005	5569	962	7864	8826
Guizhou	520000	368	285	43	1078	313	1461	1774
Yunnan	530000	427	359	298	2955	571	3468	4039
Southwestern China	/	1691	1296	2387	10491	2022	13843	15865

#### Accuracy metrics

The study implemented the metrics in different scales to demonstrate the accuracy of each provincial unit and the whole of southwestern China. Accuracy assessment of different cropland maps in Southwestern China is a problem of precision assessment in a binary classification scenario. Five metrics commonly used to evaluate the performance of machine learning as well as binary remote sensing classification are used here^60^: user accuracy (, known as precision), producer accuracy, (known as recall or sensitivity), F1-score, overall accuracy (${OA}$), and Matthew’s correlation coefficient (${MCC}$). The MCC encompasses true positives (TP), true negatives (TN), false positives (FP), and false negatives (FN) and is generally regarded as a balanced indicator. It remains applicable even when there is a significant disparity in the sample sizes of the two categories. A high score is only produced when predictions achieve satisfactory outcomes across all four categories of the confusion matrix (TP, TN, FN, and FP). According to the study of Chicco and Jurman^61^, the MCC produces a more reliable statistical rate, which makes a high score, especially in the binary classifications and their confusion matrices. We also adjusted the accuracy using the methods proposed by Olofsson, *et al*.^60^.

The formulas are shown below:

1 $${Overall\; Accuracy}\,({OA})=\frac{{TP}+{TN}}{{TP}+{FP}+{TN}+{FN}}$$

2 $${Producers\; Accuracy}\,({PA})=\frac{{TP}}{{TP}+{FN}}$$

3 $${User\; Accuracy}\,({UA})=\frac{{TP}}{{TP}+{FP}}$$

4 $${F1}_{{Score}}=\frac{2\times \left({PA}\times {UA}\right)}{\left({PA}+{UA}\right)}$$

5 $${MCC}=\frac{{TP}\times {TN}-{FP}\times {FN}}{\sqrt{({TP}+{FP})({TP}+{FN})({TN}+{FP})({TN}+{FN})}}$$

Where the *TP* (*True Positive*) and *TN* (*True Negative*) stand for cropland/ non-cropland samples that were correctively mapped; while the *FP* (*False Positive*) and *FN* (*False Negative*) stand for cropland/ non-cropland samples being incorrectly mapped to the other category, respectively.

### Generation of refined cropland map

**Fig. 4:**
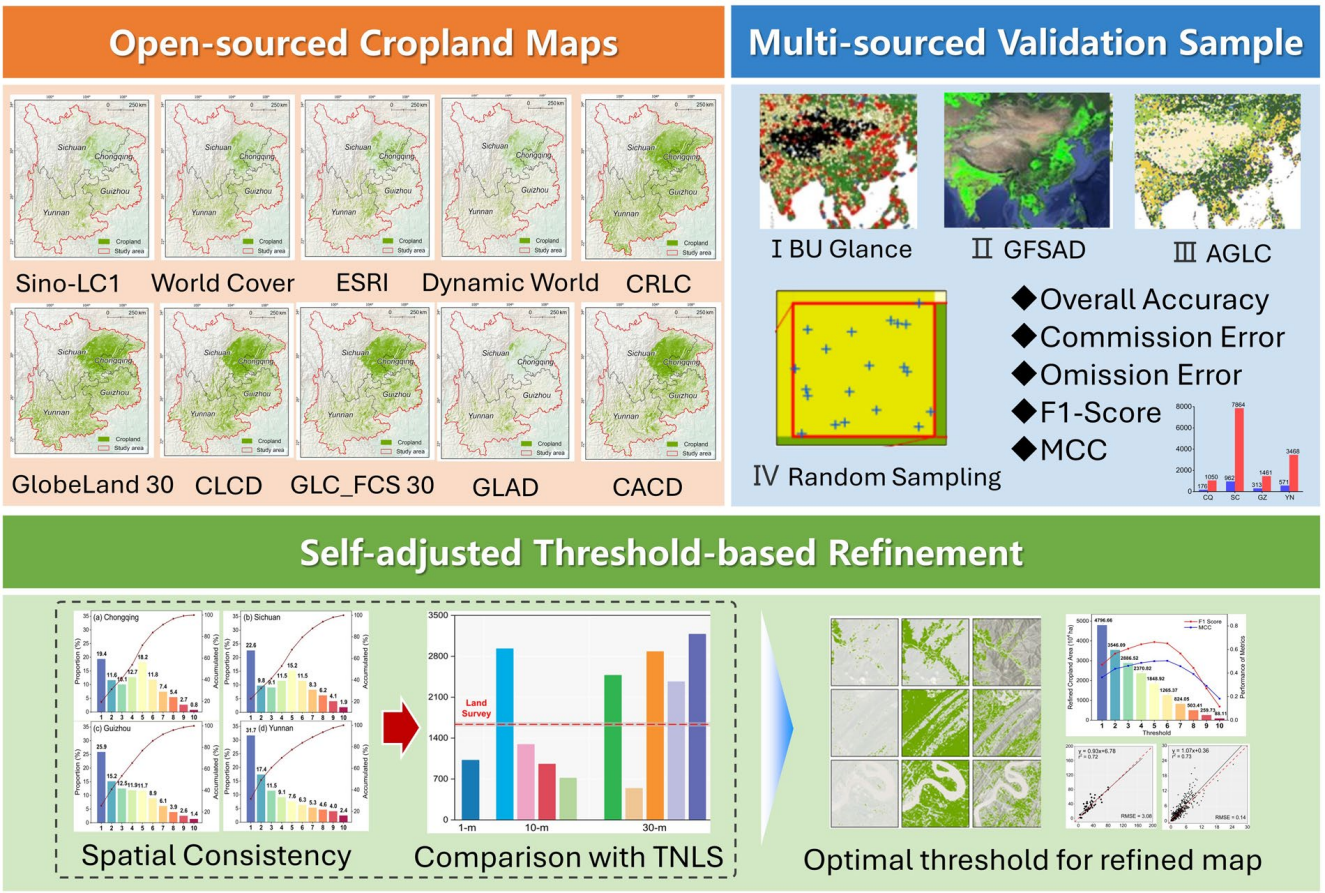
Flowchart of study.

**Fig. 5:**
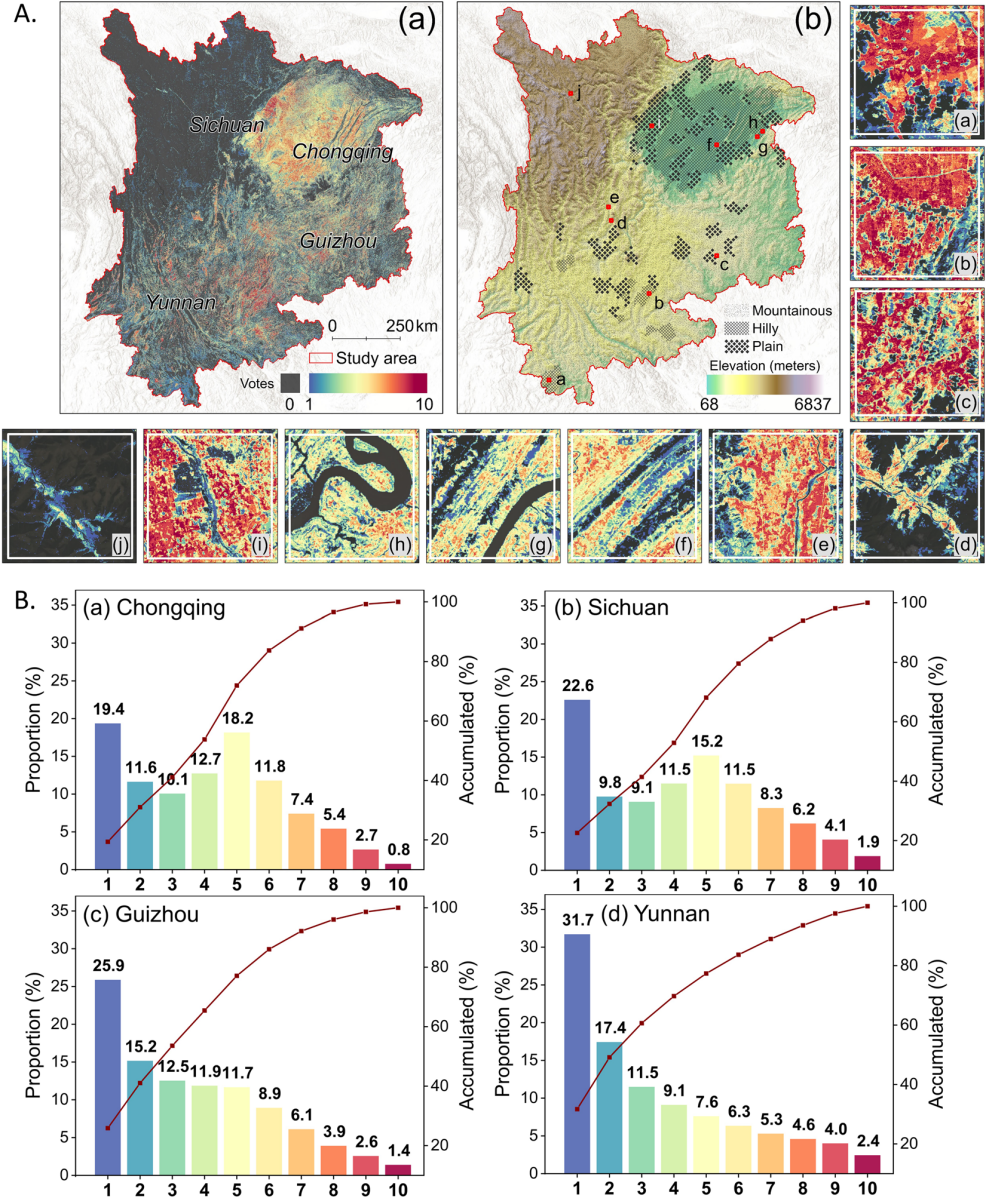
Consistency map for ten existing cropland maps in southwestern China (**A**) and statistics in four provincial levels (**B**).

We harmonized the ten existing cropland maps and generated a refined cropland map through the self-adjusted threshold method (Fig. 4). Specifically, we generated a vote map, where each pixel indicates the frequency with which it is labeled cropland among ten different cropland maps (Fig. 5). Exception for pixels not identified as cropland by any datasets (grey areas on the voting maps), the frequency values have a minimum of 1 and a maximum of 10. We ranked the frequencies from smallest to largest, used a total of 10 numbers from 1 to 10 as thresholds, and then extracted the range of cropland under the corresponding thresholds. For example, a threshold of 1 indicates that only one of the ten datasets is considered to be cropland, in which case the generated cropland map is noted as ${{Map}}_{{threshold\_}01}$. Similarly, a threshold of 10 indicates that all ten datasets are cropland, in this case, the generated cropland map is noted as ${{Map}}_{{threshold\_}10}$. By extracting different thresholds, we generated ten cropland maps (i.e., ${{Map}}_{{threshold\_}01},{{Map}}_{{threshold\_}02},\ldots ,{{Map}}_{{threshold\_}10}$) that synthesized the consistency of the ten datasets. Furthermore, the cropland area, overall accuracy, and F1 scores of the individual cropland maps derived from refined maps with different thresholds were calculated, and histograms were drawn.

**Fig. 6:**
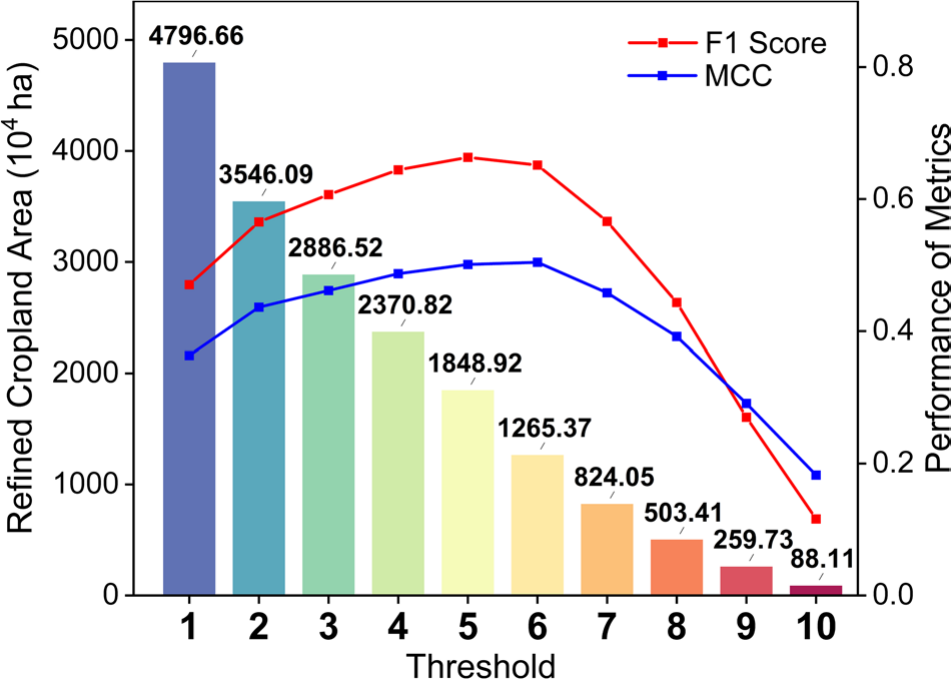
Cropland area derived from refined map and performance of metrics by thresholds. The red line and blue line represent the F1 Score and MCC.

According to the principle that the F1 score is the highest and bias between the mapped area with statistics from TNLS being the lowest, the optimal cropland map generated by the corresponding frequency thresholds (${{Map}}_{{threshold\_refined}}$) is determined to be the refined cropland map. The metric performance is shown in Fig. 6.

### Cropland area comparison at multiple scales

#### Area-based comparison

In this study, the cropland area derived from each of the ten cropland datasets was calculated at three administrative levels: the whole of southwestern China, provincial, and district scale, respectively. The administrative boundary file comes from the Resource and Environment Science and Data Canter, Chinese Academy of Sciences (https://www.resdc.cn/). Then, they were compared with the census data from the Third National Land Survey (TNLS) (https://gtdc.mnr.gov.cn). It was worth noting that due to the special administrative organization setup of Chongqing as a municipality directly under the central government (equivalent to a provincial administrative unit), the district-level administrative entities under its jurisdiction (e.g., Wanzhou District of Chongqing) are treated as prefectural-level municipalities to be compared with prefectural-level municipalities in other provinces. Two metrics, including *R*^2^ and Root Mean Square Error (*RMSE*), were characterized to measure the correlation between the mapped area and statistics, and the formulas are listed below.

6 $${R}^{2}=1-\frac{\mathop{\sum }\limits_{i=1}^{n}{\left({x}_{i}-{y}_{i}\right)}^{2}}{\mathop{\sum }\limits_{i=1}^{n}{\left({y}_{i}-\bar{y}\right)}^{2}}$$

7 $${RMSE}=\sqrt{\frac{\mathop{\sum }\limits_{i=1}^{n}{\left({x}_{i}-{y}_{i}\right)}^{2}}{n}}$$

where the ${y}_{i}$ and $\bar{y}$ represented the TNLS area and the mean value of TNLS, the ${x}_{i}$ indicated the mapped area, and the $n$ showed the number of administrative units, respectively.

#### Spatial extent comparison

The spatial distribution of cropland in southwestern China is heterogeneous, which means that consistency in the overall area does not necessarily lead to spatial consistency. To compare the performance of different datasets in spatial details, the study conducted a pixel-by-pixel comparison and generated a vote map for the ten cropland products. The frequency of pixels labeled cropland was performed and demonstrated as a number of votes from 1 to 10 from bad to good as to consistency. Pixels that were not labeled as cropland by any of the datasets were not considered and labeled as zero.

**Fig. 7:**
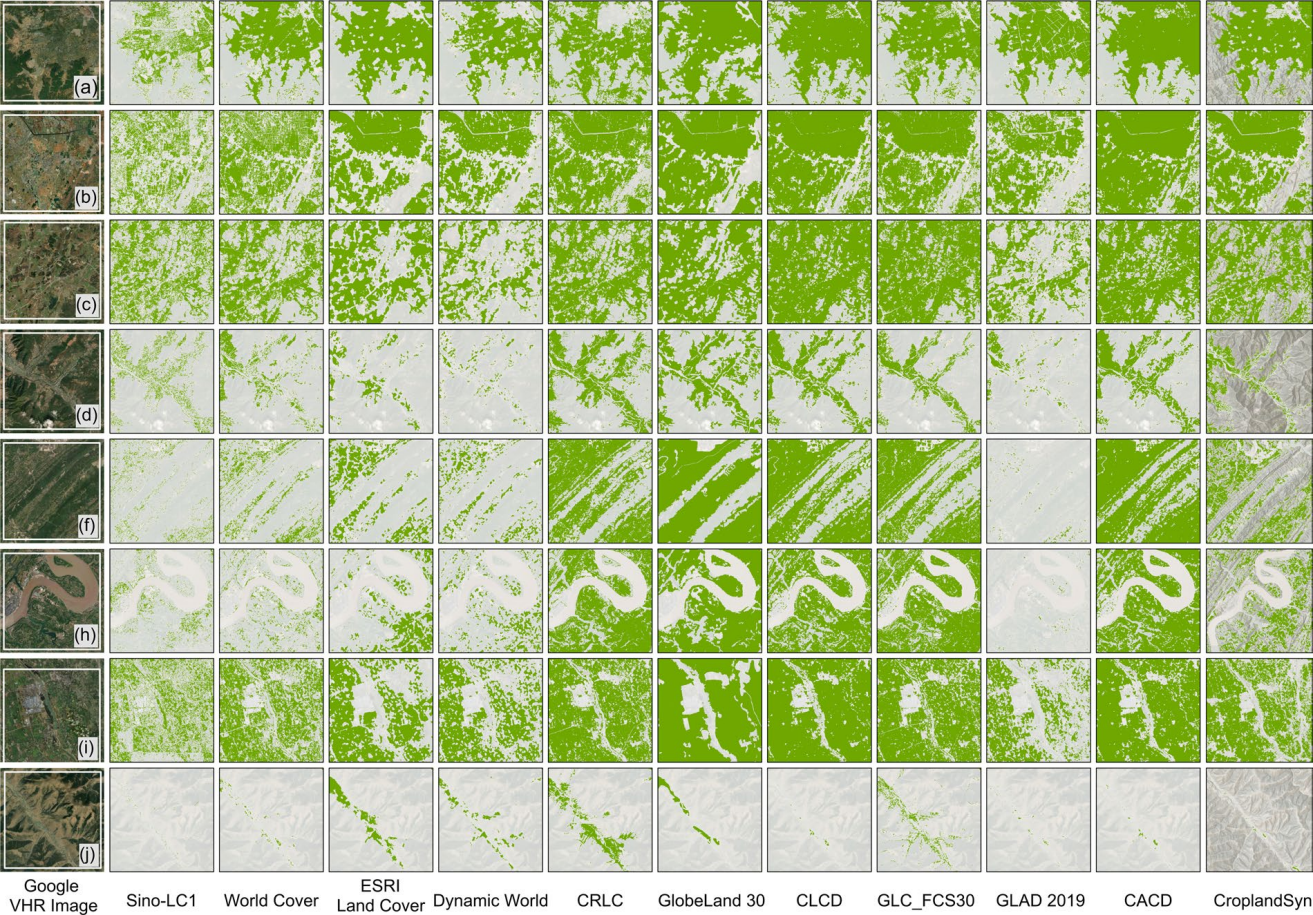
Cropland extent comparison in eight sites across southwestern China. The specific location of site (**a**–**j**) presented above can be found in Fig. 5. The hill shade derived from the Digital Elevation Model (DEM) was overlayed to each of the twelve cropland maps to demonstrate the terrain of each site, and the transparency of the shading was especially noticeable for the refined map column. The refined map in the last column originates from threshold_05, also named Cropland_syn_.

Furthermore, eight sites distributed in southwestern China (the zoomed-in view are indicated in Fig. 7) were selected in this study to demonstrate the spatial details of the cropland maps. These eight sites covered plains, hills, mountains, river valleys, and other typical landscapes in southwestern China to demonstrate that the refined map better depicts the cropland extent.

## Data Records

The refined cropland map generated based on the methods of this article is named Cropland_Syn_, depending on the optimal threshold presented in Table 2. Specifically, the threshold_05 has the highest F1 Score and is more consistent with statistical data but has a slightly lower OA than the threshold_06, which has a higher OA. The Cropland_Syn_ based on the threshold_05 significantly improved the area and extent of cropland mapping.

**Table 2:** Performance of refined map by thresholds.

Threshold	OA	PA	UA	OE	CE	F1- score	Mapped Area	MCC
Threshold_01	0.724	0.961	0.311	0.039	0.689	0.470	4796.66	0.444
Threshold_02	0.821	0.914	0.409	0.086	0.591	0.565	3546.09	0.533
Threshold_03	0.860	0.851	0.472	0.149	0.528	0.607	2886.52	0.564
Threshold_04	0.889	0.790	0.544	0.210	0.456	0.644	2370.82	0.595
Threshold_05	0.910	0.698	0.632	0.302	0.368	0.663	1848.92	0.612
Threshold_06	0.922	0.571	0.758	0.429	0.242	0.651	1265.37	0.616
Threshold_07	0.916	0.430	0.829	0.570	0.171	0.566	824.05	0.560
Threshold_08	0.905	0.296	0.885	0.704	0.115	0.443	503.41	0.479
Threshold_09	0.891	0.158	0.920	0.842	0.080	0.270	259.73	0.356
Threshold_10	0.880	0.062	0.933	0.938	0.067	0.116	88.11	0.223

The OE and CE are omission and commission errors numerically calculated by 1 minus PA and UA, respectively. The unit of cropland area and bias with land survey are in ten thousand hectares.

The vote map of inconsistency and refined cropland map in GeoTIFF format with Albers conic equal area projected coordinate system at 30-m resolution and their attached pyramid file in.ovr format are available from the figshare repository^62^. All the raster data can be loaded and edited both in script tools (such as rasterio, gdal, cartopy, etc.) and software supporting.tif format files, such as ESRI ArcGIS (https://www.esri.com/) and QGIS (https://qgis.org/).

The cropland/non-cropland samples of southwestern China in ESRI shapefile format were also shared in the repository. There are five fields in the attribute table; in addition to the latitude and longitude coordinates, the land field has values of 0 and 10, representing non-cropland and cropland samples, respectively. The Source field provides the source of the sample points. The Albers conic equal area projected coordinate system file applied to southwestern China ends with “.prj” format is also uploaded to the repository, which is available for data users in ESRI ArcGIS to reuse without self-definition.

## Technical Validation

Two methods were used to validate of the resultant maps, including the sample-aided accuracy assessment and cross-comparison with the existing ten cropland maps.

### Accuracy assessments of cropland maps

To quantitatively characterize the accuracies of the ten existing cropland maps and the refined map at multi-administrative scales, we used the previously constructed sample dataset to validate them in Southwestern China and at different provincial administrative scales.

**Fig. 8:**
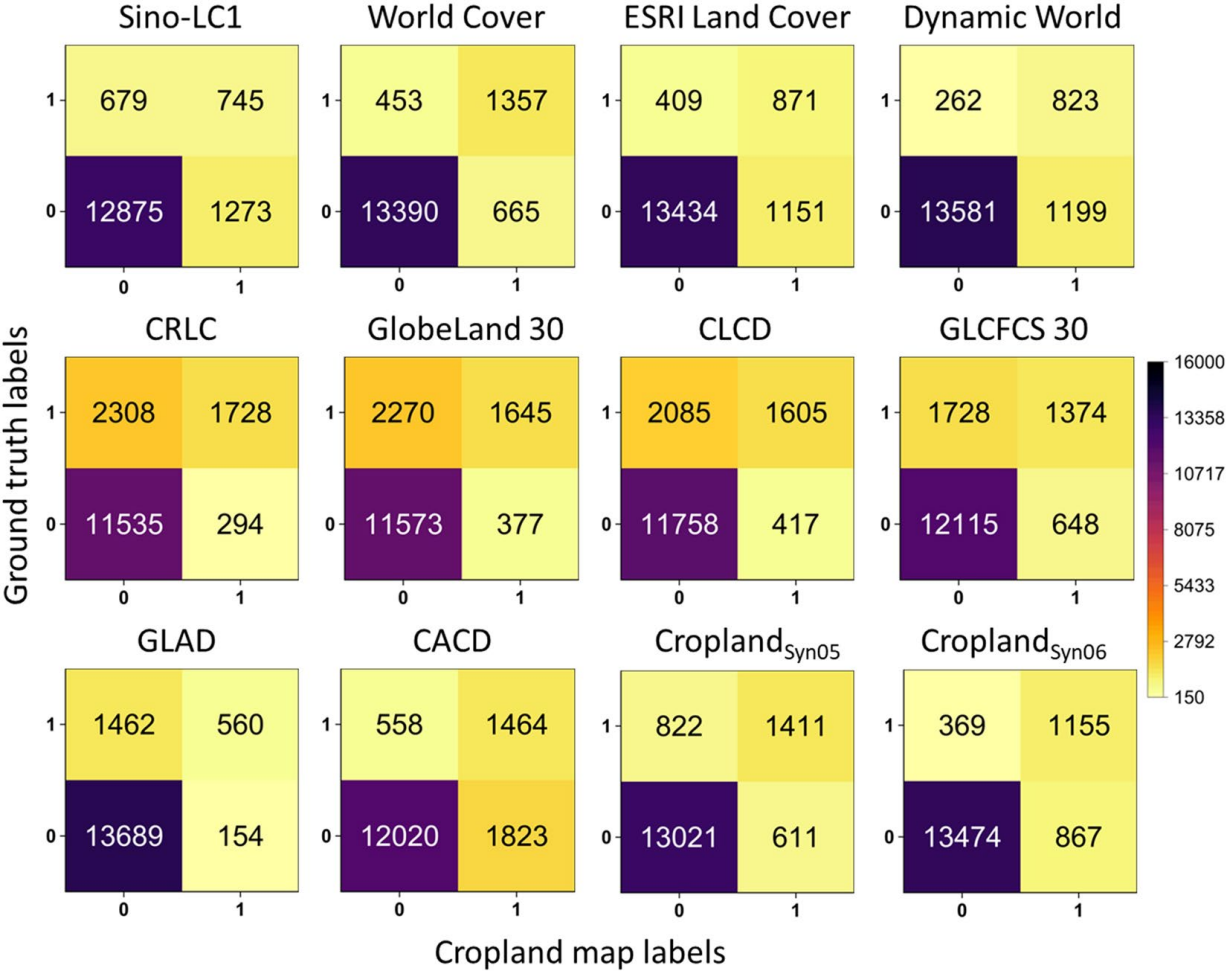
Confusion matrix of cropland maps used in the study of southwestern China.

**Fig. 9:**
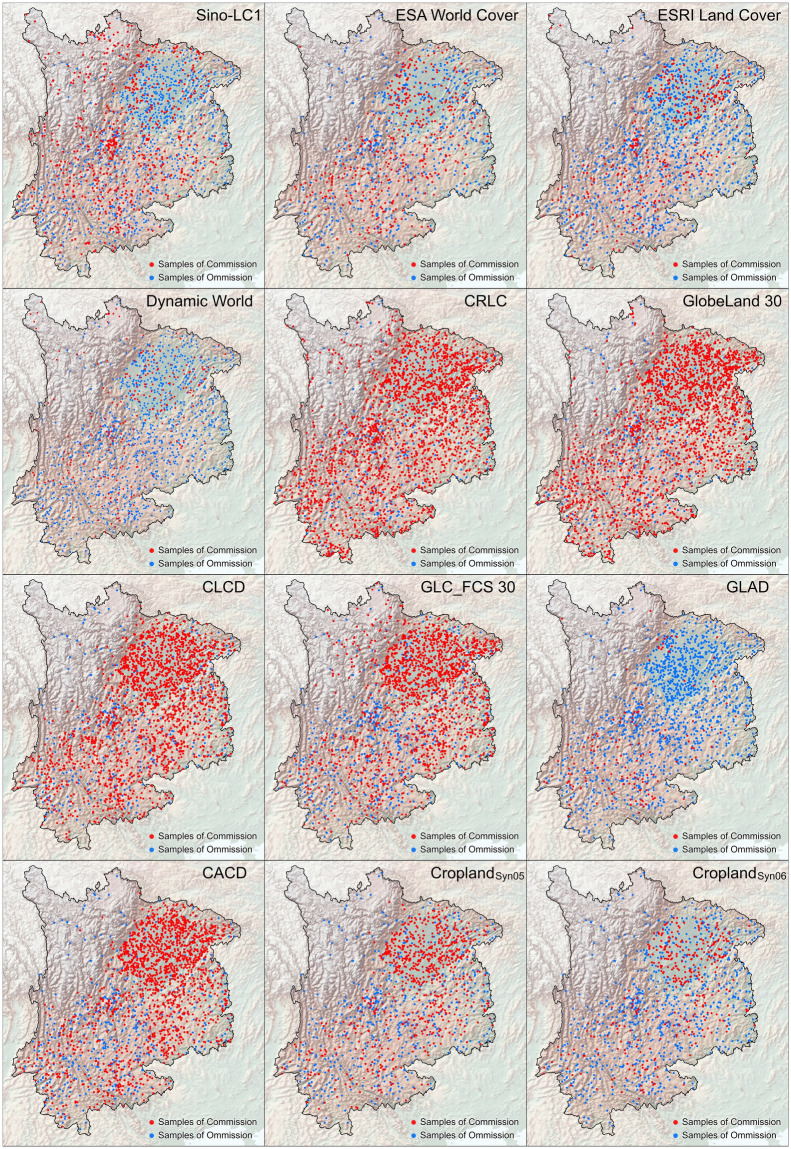
Samples of commission and omission for cropland maps in southwestern China.

The results showed that the 30-m refined cropland map (in which the threshold_05 was renamed as Cropland_Syn_ in Data Records) based on the thresholds of the vote map ranked among the highest in accuracy (generally with an overall accuracy higher than 0.80) at both the provincial administrative scales and the southwestern China regarding overall accuracy, F1 score, and MCC values (Fig. 8 and Table S2). This is also supported by the sample-based error distribution in Fig. 9. It outperformed most of the ten existing products (especially all five datasets with the same 30-m spatial resolution, where the accuracy of these datasets is even lower than average). It was only surpassed by the WorldCover, which has a higher spatial resolution of 10 m.

### Comparisons with existing cropland maps and land survey data

Figure 2 shows the spatial distribution of 12 cropland maps in southwestern China. To avoid the difference in visual effects caused by spatial resolution, the nearest-neighbor method was used to resample cropland maps with spatial resolution of 10- or 1 m to 30 m. Overall, the refined cropland map based on threshold_05 improved both the overestimation in cropland area for the existing 30-m data (mainly due to misclassification errors) and the underestimation of cropland in the 10-m and 1-m data (mainly due to omission errors). Specifically, this is evidenced by the spatial extent comparisons performed on the eight sites illustrated in Fig. 7. For the plain areas (c, i in Fig. 7), the optimized cropland maps provided cropland mapping that was more consistent with the actual distribution. In hilly areas (b,f,h in Fig. 7), the misclassification of the other 30-m cropland maps can be substantially improved.

**Fig. 10:**
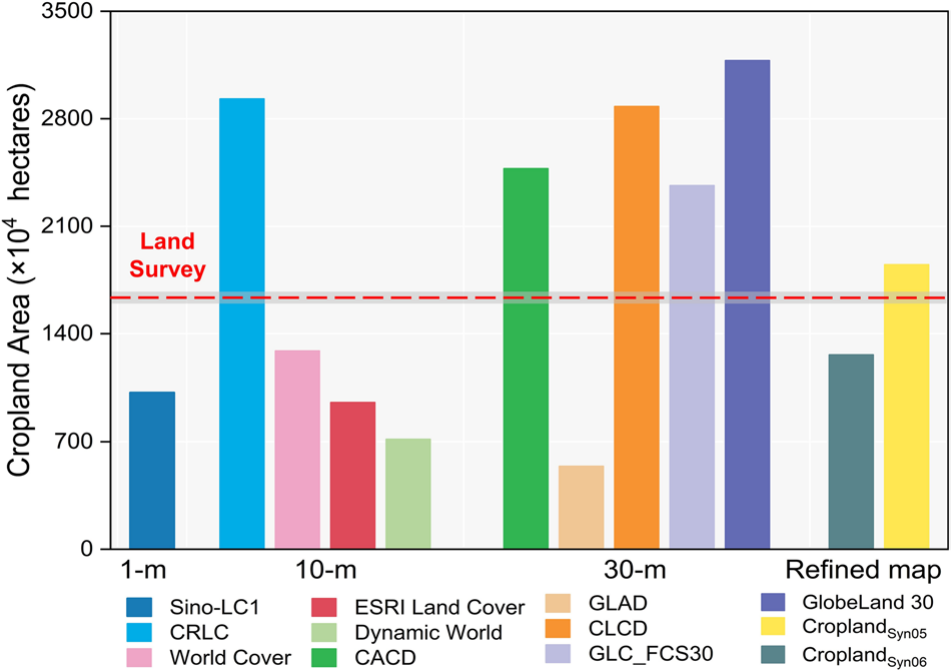
Cropland area derived from cropland maps used in the study of southwestern China. The red reference dashed line parallel to the X-axis indicates the total cropland area of the Third National Land Survey (TNLS) Closer distance to the dashed line in the Y-axis direction indicates that the dataset is closer to the TNLS cropland area.

**Fig. 11:**
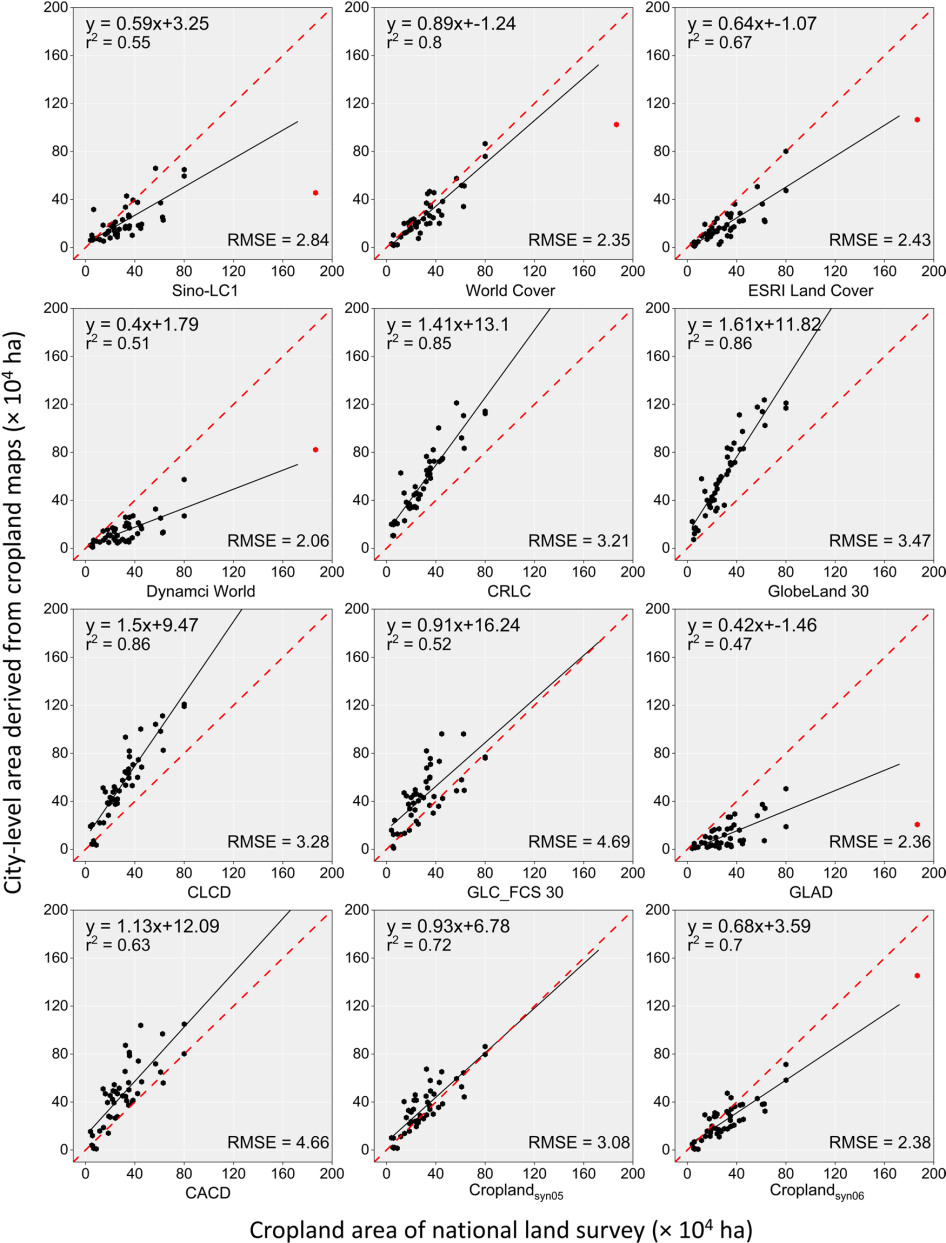
Prefecture city-level comparison between mapped area and cropland area from TNLS. The red dots in the figure represent data from Chongqing City, which has been excluded from the comparison due to its exceptional administrative level.

**Fig. 12:**
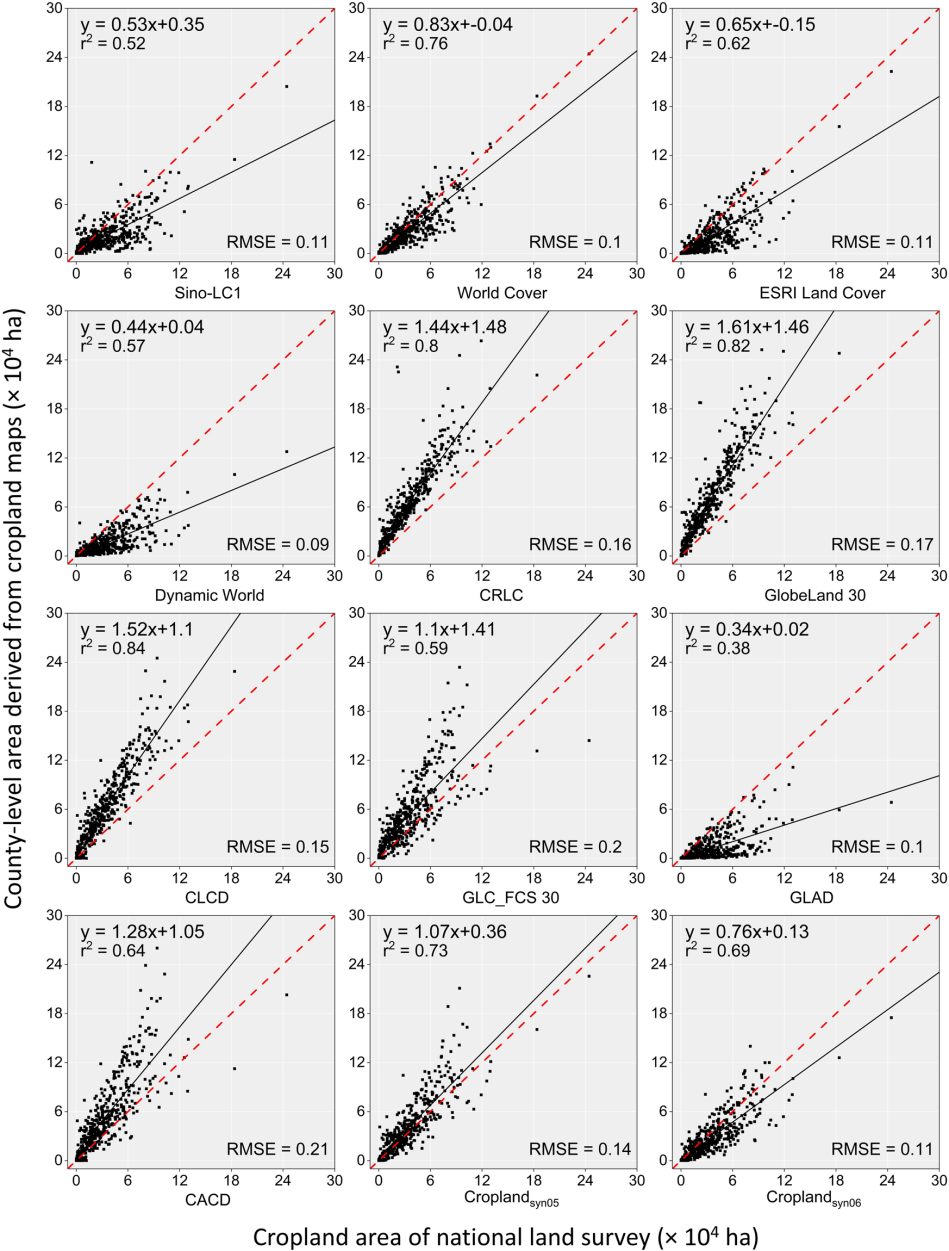
County-level comparison between mapped area and cropland area from TNLS.

The mapped area of each cropland map was further compared with the statistics at different administrative levels from the TNLS, which has been recognized as the most precise source of land area data in China^63^. In general, cropland maps with higher spatial resolution (1-m, 10-m) tended to underestimate the cropland area in Chinese regions compared to the area published by TNLS (red dashed line in Fig. 10). In contrast, the 30-m cropland maps tended to overestimated the area of croplands. There are exceptions, however, with the 10-m CRLC and the 30-m GLAD showing the opposite trends compared to other cropland maps with the same spatial resolution, respectively. The refined maps for thresholds 5 and 6 showed a much smaller gap from the red line in the figure, which is significantly superior to the other nine pre-existing cropland maps except for World Cover, and the differences from World Cover were extremely small. In addition, the area of the 12 cropland maps was compared at the prefecture level (Fig. 11) and county level (Fig. 12), respectively. The scattered points of the refined cropland maps were more centrally distributed along the 1:1 line than the overestimation and underestimation of the existing maps. This suggested that the refined map reduces both the omission error of high spatial resolution maps and the commission error of low spatial resolution maps, approaching the accuracy of the NLSD regarding cropland area.

### Uncertainty analysis

Several uncertainties may exist in the dataset. First, for the generation of vote maps, pixels labeled as non-cropland with pixel values equal to zero may have been caused by omission errors in the corresponding cropland product. For example, cropland maps refined using the thresholding method may lose a partition of accuracy due to the omission errors in some of the mountainous areas in Fig. 7(j). It follows that there may be some pixels that are truly cropland not identified as cropland by any of the datasets, further contributing to the omission error in the refined cropland map. In future work, adding training samples in areas of high inconsistency according to the vote map to improve the accuracy in regions with cropland mapping difficulties (i.e., hilly and mountainous regions in Fig. 5) is a possible solution^64^.

Second, the study performed resampling to harmonize the spatial resolution of the ten available cropland data products, which has to some extent weakened the ability of the 1-m or 10-m spatial resolution datasets to depict cropland details (See in data pre-processing). Although cropland mapping is inherently difficult in an area with such diverse land cover types and fragmented parcels, the refined map produced in this study still offered a more significant improvement in accuracy and for cropland area (Fig. 7 and Fig. 8). This study also demonstrates the feasibility of generating optimized new data from existing data in an era when open-sourced cropland maps are becoming increasingly abundant^65–67^. For further improvements in data fusion, it is possible to generate higher-precision cropland maps by optimizing and integrating datasets based on geographic subdivisions and data-driven alogrithms^68,69^.

Third, the study has analysed ten recently released cropland maps since 2021. However, only the year 2020 was considered for the comparison. Most of these maps (including World Cover, ESRI Land Cover, Dynamic Word, CLCD, GLC_FCS 30, CACD, and GLAD) can reflect long-term cropland dynamics^23^. Therefore, the following work could be focused on optimizing time-series cropland maps with a time-series validation sample set to meet the demands of dynamic cropland monitoring better.

## Usage Notes

Cropland maps play an indispensable role in guiding agricultural land management. However, in an era of continuous enrichment of open-source data, inconsistencies across datasets hinder our understanding of agricultural land systems’ processes, patterns, and responses to anthropogenic disturbances. Compared to the existing ten cropland datasets, our data-driven refined map (CroplandSyn) shows higher consistency with official land survey data and greater accuracy. Additionally, the shared sample set of this work facilitates quality assessment and continuous refinement of cropland maps in Southwest China.

### Supplementary information

Supplementary Materials

## Data Availability

JavaScript codes of the Earth Engine repository used to generate the cropland maps and metrics for accuracy evaluation are shared and available from the figshare repository. Python codes for raster pyramid building are also provided. The software and modules used in this study include Origin 2023b, ArcGIS Pro 3.1, Python 3.7, gma 1.1.5, and ArcPy 3.1. The very high spatial resolution (VHR) Google Earth images are accessible by the ArcGIS Web Map Tile Service (WTMS) service.
