# Supplementary material for: Validation and refinement of cropland map in southwestern China by harnessing ten contemporary datasets
Source: Sci Data. 2024 Jun 22;11:671. doi: 10.1038/s41597-024-03508-5 (PMC11193745; doi:10.1038/s41597-024-03508-5)
Supplement: Supplementary file 1 — Supplementary Materials [file 41597_2024_3508_MOESM1_ESM.docx]

**Table of contents**

[Table S1 Summary of ten cropland maps used in this study. 2](#_Toc166765503)

[Table S2 Metrics performance of twelve cropland maps in four provinces of southwestern China. 5](#_Toc166765504)

# **Table S1 Summary of ten cropland maps used in this study**.

Note: (1) The ordinal numbers in the list are arranged in ascending order by spatial resolution of the dataset, and for datasets with the same resolution then in ascending order by time of publication. (2) The spatial resolution of SinoLC-1 is approximately 1.07 m due to applying level 18 optical images collected from Google Earth. (3) The Landsat Analysis Ready Data (ARD) used in GLAD is a senior product created by USGS to make Landsat satellite imagery more easily accessible and usable for scientific research and applications, like land cover mapping. (4) The table’s current time span and cropland code indicate the time range covered by the dataset up to the completion of this manuscript and the code for cropland class in multi-category datasets, respectively.

| Num. | Cropland datasets | Data Source | Spatial Resolution | Definition of Cropland by producer | Current time span | Cropland  code | Publish year | Source |
| --- | --- | --- | --- | --- | --- | --- | --- | --- |
| 1 | SinoLC-1 | VHR images from  Google Earth | ~1.07 m | Including paddy field, dry land, irrigated land, orchard, tea and rubber plantations. | 2020 | 5 | 2023 | Li, et al. ^45^ |
| 2 | World Cover | Sentinel-1,  Sentinel-2 | 10 m | Land covered with annual cropland that is sowed/planted and harvestable at least once within the 12 months after the sowing/planting date. | 2017-2022 | 40 | 2021 | Zanaga, et al. ^46^ |
| 3 | ESRI  Land Cover | Sentinel-2 | 10 m | Human planted or plotted cereals, grasses, and crops not at tree height; examples: corn, wheat, soy, fallow plots of structured land. | 2017-2022 | 5 | 2021 | Karra, et al. ^47^ |
| 4 | Dynamic World | Sentinel-1,  Sentinel-2 | 10 m | Human planted or plotted cereals, grasses, and crops. | 2015-Now | 4 | 2022 | Brown, et al. ^48^ |
| 5 | CRLC | Sentinel-2 | 10 m | A category parallel to the other seven types of land cover including forest, grass/shrubland, and wetland etc. No specific definition by data producer. | 2020 | 1 | 2023 | Liu, Zhong, Ma, Zhao and Zhang ^49^ |
| 6 | GlobeLand 30 | Landsat 5/7/8 (TM5, ETM+, OLI),  HJ-1, GF-1 | 30 m | Lands used for cultivating crops and land mainly planted with crops rarely with economic crops. | 2000, 2010, 2020 | 10 | 2021 | Chen, et al. ^50^ |
| 7 | CLCD | Landsat 8 OLI | 30 m | Cropland (paddy rice, greenhouse, others), orchard, managed grasslands, temporally bare croplands. | 1985-2022 | 1 | 2021 | Yang and Huang ^51^ |
| 8 | GLC_FCS 30 | Landsat 8 OLI | 30 m | Rainfed/irrigated cropland, herbaceous cover, tree or shrub cover (orchard). | 1985-2020,  every 5 years | 10,11,12,20 | 2021 | Zhang, et al. ^70^ |
| 9 | GLAD | Landsat ARD | 30 m | The land used for annual and perennial herbaceous crops for human consumption, forage (including hay) and biofuel. | 2000-2019,  4-year-composite | 17 | 2022 | Potapov, et al. ^55^ |
| 10 | CACD | Landsat 5/7/8 | 30 m | The piece of land with 0.09 ha in minimum, sowed or planted and harvestable at least once within the 12 months after the sowing or planting date. | 1986-2021 | 1 | 2023 | Tu, et al. ^54^ |

# Table S2 Metrics performance of twelve cropland maps in four provinces of southwestern China.

| Province | Metric | | CroplandSyn05 | | Sino-LC1 | | World Cover | | ESRI Land Cover | | Dynamic World | | CRLC | | GlobeLand 30 | | CLCD | | GLC_FCS 30 | | GLAD | | CACD | | Mean | |
| --- | --- | --- | --- | --- | --- | --- | --- | --- | --- | --- | --- | --- | --- | --- | --- | --- | --- | --- | --- | --- | --- | --- | --- | --- | --- | --- |
| Chongqing | Overall Accuracy | | 0.844 | | 0.859 | | 0.892 | | 0.855 | | 0.889 | | 0.660 | | 0.654 | | 0.691 | | 0.680 | | 0.869 | | 0.700 | | 0.781 | |
|  | PA | | 0.750 | | 0.182 | | 0.675 | | 0.489 | | 0.520 | | 0.955 | | 0.922 | | 0.861 | | 0.843 | | 0.138 | | 0.877 | | 0.656 | |
|  | UA | | 0.473 | | 0.533 | | 0.691 | | 0.513 | | 0.683 | | 0.304 | | 0.285 | | 0.316 | | 0.299 | | 0.808 | | 0.316 | | 0.475 | |
|  | F1 Score | | 0.580 | | 0.271 | | 0.634 | | 0.488 | | 0.562 | | 0.461 | | 0.435 | | 0.459 | | 0.439 | | 0.208 | | 0.464 | | 0.455 | |
|  | MCC | | 0.510 | | 0.252 | | 0.581 | | 0.408 | | 0.514 | | 0.413 | | 0.375 | | 0.386 | | 0.360 | | 0.279 | | 0.397 | | 0.407 | |
| Sichuan | Overall Accuracy | | 0.922 | | 0.887 | | 0.939 | | 0.916 | | 0.924 | | 0.865 | | 0.851 | | 0.852 | | 0.858 | | 0.913 | | 0.856 | | 0.889 | |
|  | PA | | 0.715 | | 0.364 | | 0.708 | | 0.462 | | 0.484 | | 0.888 | | 0.846 | | 0.827 | | 0.762 | | 0.223 | | 0.806 | | 0.644 | |
|  | UA | | 0.622 | | 0.494 | | 0.778 | | 0.701 | | 0.767 | | 0.476 | | 0.445 | | 0.447 | | 0.458 | | 0.854 | | 0.455 | | 0.591 | |
|  | F1 Score | | 0.665 | | 0.419 | | 0.715 | | 0.532 | | 0.570 | | 0.614 | | 0.576 | | 0.572 | | 0.559 | | 0.371 | | 0.572 | | 0.561 | |
|  | MCC | | 0.623 | | 0.363 | | 0.686 | | 0.507 | | 0.555 | | 0.586 | | 0.540 | | 0.532 | | 0.508 | | 0.422 | | 0.528 | | 0.532 | |
| Guizhou | Overall Accuracy | | 0.871 | | 0.847 | | 0.905 | | 0.864 | | 0.864 | | 0.763 | | 0.771 | | 0.764 | | 0.777 | | 0.848 | | 0.795 | | 0.824 | |
|  | PA | | 0.674 | | 0.469 | | 0.733 | | 0.391 | | 0.278 | | 0.861 | | 0.805 | | 0.782 | | 0.666 | | 0.183 | | 0.723 | | 0.597 | |
|  | UA | | 0.620 | | 0.584 | | 0.755 | | 0.709 | | 0.826 | | 0.419 | | 0.428 | | 0.401 | | 0.403 | | 0.759 | | 0.429 | | 0.576 | |
|  | F1 Score | | 0.647 | | 0.520 | | 0.731 | | 0.503 | | 0.427 | | 0.563 | | 0.557 | | 0.533 | | 0.509 | | 0.318 | | 0.546 | | 0.532 | |
|  | MCC | | 0.569 | | 0.434 | | 0.677 | | 0.458 | | 0.436 | | 0.479 | | 0.462 | | 0.432 | | 0.391 | | 0.339 | | 0.443 | | 0.465 | |
| Yunnan | Overall Accuracy | 0.946 | | 0.864 | | 0.919 | | 0.914 | | 0.894 | | 0.815 | | 0.834 | | 0.858 | | 0.876 | | 0.898 | | 0.870 | | 0.881 | |  |
|  | PA | 0.664 | | 0.381 | | 0.694 | | 0.447 | | 0.371 | | 0.802 | | 0.781 | | 0.774 | | 0.566 | | 0.434 | | 0.588 | | 0.591 | |  |
|  | UA | 0.749 | | 0.534 | | 0.721 | | 0.708 | | 0.749 | | 0.419 | | 0.449 | | 0.499 | | 0.563 | | 0.734 | | 0.535 | | 0.606 | |  |
|  | F1 Score | 0.704 | | 0.445 | | 0.707 | | 0.548 | | 0.496 | | 0.551 | | 0.570 | | 0.607 | | 0.564 | | 0.546 | | 0.560 | | 0.573 | |  |
|  | MCC | 0.660 | | 0.377 | | 0.660 | | 0.509 | | 0.479 | | 0.489 | | 0.504 | | 0.544 | | 0.492 | | 0.514 | | 0.485 | | 0.519 | |  |
| Southwestern China | Overall Accuracy | 0.955 | | 0.873 | | 0.929 | | 0.901 | | 0.909 | | 0.833 | | 0.814 | | 0.830 | | 0.843 | | 0.955 | | 0.834 | | 0.880 | |  |
|  | PA | 0.698 | | 0.382 | | 0.672 | | 0.444 | | 0.388 | | 0.858 | | 0.839 | | 0.813 | | 0.698 | | 0.556 | | 0.630 | | 0.635 | |  |
|  | UA | 0.632 | | 0.523 | | 0.750 | | 0.680 | | 0.759 | | 0.428 | | 0.420 | | 0.435 | | 0.443 | | 0.277 | | 0.724 | | 0.552 | |  |
|  | F1 Score | 0.663 | | 0.433 | | 0.708 | | 0.528 | | 0.530 | | 0.570 | | 0.554 | | 0.562 | | 0.536 | | 0.409 | | 0.552 | | 0.550 | |  |
|  | MCC | 0.612 | | 0.372 | | 0.670 | | 0.491 | | 0.513 | | 0.527 | | 0.502 | | 0.508 | | 0.466 | | 0.428 | | 0.487 | | 0.507 | |  |
